# Extracellular Membrane Vesicles of *Escherichia coli* Induce Apoptosis of CT26 Colon Carcinoma Cells

**DOI:** 10.3390/microorganisms12071446

**Published:** 2024-07-17

**Authors:** Yao Jiang, Jing Ma, Yuqing Long, Yuxi Dan, Liaoqiong Fang, Zhibiao Wang

**Affiliations:** 1 State Key Laboratory of Ultrasound in Medicine and Engineering, College of Biomedical Engineering, Chongqing Medical University, Chongqing 400016, China; jyao@stu.cqmu.edu.cn (Y.J.); cqmumj@stu.cqmu.edu.cn (J.M.); long_yuqing@stu.cqmu.edu.cn (Y.L.); cqmudyx@stu.cqmu.edu.cn (Y.D.); 2 Chongqing Key Laboratory of Biomedical Engineering, Chongqing Medical University, Chongqing 400016, China; 3 National Engineering Research Center of Ultrasound Medicine, Chongqing 401121, China

**Keywords:** *E. coli*-OMVs, CT26, apoptosis

## Abstract

*Escherichia coli* (*E. coli*) is commonly utilized as a vehicle for anti-tumor therapy due to its unique tumor-targeting capabilities and ease of engineering modification. To further explore the role of *E. coli* in tumor treatment, we consider that *E. coli* outer membrane vesicles (*E. coli*-OMVs) play a crucial role in the therapeutic process. Firstly, *E. coli*-OMVs were isolated and partially purified by filtration and ultracentrifugation, and were characterized using techniques such as nanoparticle tracking analysis (NTA), transmission electron microscopy (TEM) and Western Blot (WB). The obtained extracellular nanoparticles, containing OMVs, were found to inhibited the growth of CT26 tumor in mice, while the expression of Bax protein was increased and the expression of Bcl-2 protein decreased. In vitro experiments showed that *E. coli*-OMVs entered CT26 cells and inhibited cell proliferation, invasion and migration. In addition, in the presence of *E. coli*-OMVs, we observed an increase in apoptosis rate and a decrease in the ratio of Bcl-2/Bax. These data indicate that *E. coli*-OMVs inhibits the growth of CT26 colon cancer by inducing apoptosis of CT26 cells. These findings propose *E. coli*-OMVs as a promising therapeutic drug for colorectal cancer (CRC), providing robust support for further research in related fields.

## 1. Introduction

Colorectal cancer (CRC), as the third most common cancer globally with continuously increasing incidence and mortality rates, is influenced by various factors such as lifestyle, environment, and genetics [1,2]. Currently, the primary treatment approach for CRC remains surgery, supplemented by adjuvant radiotherapy and chemotherapy [3]. However, cancer treatment still faces great challenges due to difficulties such as the depth of the tumor tissue location and the tolerance of tumor cells to chemoradiotherapy.

In recent years, bacterial therapy for tumors has become a research hotspot. Facultative anaerobic bacteria and obligate anaerobic bacteria have attracted significant attention due to their tumor-targeting specificity and cytotoxicity. They tend to accumulate and proliferate at the tumor site, thereby inhibiting tumor growth. Different bacteria exhibit distinct anti-tumor mechanisms in the tumor microenvironment [4]. For example, Salmonella directly exerts anti-tumor effects by inducing apoptosis and autophagy [5,6]. Additionally, Listeria enhances reactive oxygen species (ROS) levels to suppress tumor growth [7]. Furthermore, Clostridium secretes bacterial toxins, inducing the release of various cytokines and chemokines at the infection site, thereby promoting tumor elimination [8]. Bacteria have the advantage of being easily genetically modified and bioengineered, meeting clinical needs for the delivery of anticancer agents. However, as anticancer drugs, bacteria still face challenges such as low targeting efficiency and the toxic side effects of bacterial toxins on the body. These potential factors hinder the application of bacteria in cancer treatment. Thus, achieving a balance between target specificity and side effects remains key to bacterial cancer therapy.

Outer membrane vesicles (OMVs) are nano-sized spherical vehicles formed by lipid bilayer membranes released by bacteria, with a diameter ranging from 20 to 200 nm [9]. Due to their small size and inability for autonomous reproduction, OMVs exhibit better biological safety and enhance drug delivery capabilities compared to the bacteria themselves [10]. Research has found that most OMVs are produced by controlled bubbling of the bacterial outer membrane, carrying the outer membrane components and periplasmic proteins of the parent cell. Consequently, they exhibit similarities in function to the bacteria [11]. The previous research demonstrated that membrane vesicles derived from Bifidobacterium inhibited the growth of triple-negative breast cancer by inducing apoptosis [12]. However, whether Gram-negative bacteria inhibit tumor growth through the induction of apoptosis has not been investigated.

Apoptosis plays a central role in the occurrence and development of cancer, serving as a crucial self-regulatory mechanism for maintaining cellular homeostasis. The pathways involving primarily include death receptor-mediated and mitochondria-mediated pathways, both of which ultimately induce the activation of caspase enzymes [13]. The Bcl-2 protein family plays a crucial role in regulating apoptosis, where Bax and Bcl-2 can individually form heterodimers or interact with each other to form heterodimers [14]. The expression levels of these proteins are directly related to the regulation of cell apoptosis [15].

In this study, we isolated extracellular nanoparticles containing OMVs from *Escherichia coli* (*E. coli*) BL21, aiming to explore their in vitro and in vivo anti-tumor effects on CT26 colon cancer and investigate their potential mechanisms. Our results show that *E. coli*-OMVs are not only a promising anticancer drug but also a novel therapeutic agent targeting the cell apoptosis pathway for treating CRC.

## 2. Materials and Methods

All animal experimental protocols were approved by the Ethics Committee of Chongqing Medical University. Approval number IACUC-CQMU-2023-0237.

### 2.1. Cells Lines, Culture and Treatment

*E. coli* BL21 was purchased from Weidi Biotechnology (Shanghai, China) and CT26 murine colon adenocarcinoma was purchased from Boster Biological Technology (Pleasanton, CA, USA). *E. coli* was cultured in Luria–Bertani (LB) medium (Hopebio, Qingdao, China, HB0128, HB0129) and CT26 cells were cultured in Roswell Park Memorial Institute (RPMI) 1640 (Biosharp, Hefei, China, BL303A) containing 10% fetal bovine serum (FBS) (Clark Bioscience, Shanghai, China, FB-15011) at 37 °C and 5% CO_2_.

### 2.2. Reagents and Drugs

A 0.45 μm and 0.22 μm vacuum filter (Merck Millpore, Darmstadt, Germany, SLHV033RB; SLGP033RB), an ultrafiltration centrifuge tube with a molecular weight cutoff (MWCO) of 100 kDa (Merck Milllpore, UFC910024), PKH67 (Umibio, Shanghai, China, UR52303), Hoechst 33342 (Beyotime, Shanghai, China, C1022), Cell Counting Kit-8 (CCK8, Solarbio, Beijing, China, CA1210), an Annexin V/PI assay kit (Elabscience, Wuhan, China, E-CK-A211), a mitochondrial membrane potential assay kit (Beyotime, C2003S), a RIPA lysis buffer (Solarbio, R0010), TBST (Solarbio, T1082), Goat Anti-Rabbit IgG H&L (HRP) (Abcam, Cambridge, MA, USA, ab205718), enhanced chemiluminescence (ECL) reagents (Beyotime, P0018S), Caspase-3 and Caspase-9 activity assay kits (Beyotime, C1115; C1157).

### 2.3. Extraction of E. coli-OMVs

Cells from a single colony of *E. coli* were cultivated in shake flasks with LB broth until the OD_600_ value reached 1.0. Bacteria cells were removed by centrifugation (5000× *g*, 15 min, 4 °C) and the resulting supernatant was passed through filters (0.45 and 0.22 μm). The filtrate was concentrated using ultrafiltration centrifuge tube with a molecular weight cutoff (MWCO) of 100 kDa and then centrifuged (150,000× *g*, 3 h, 4 °C) twice. The obtained nanoparticles were stored at −80 °C for subsequent experiments. The protein concentration of nanoparticles was determined using the BCA protein assay kit.

### 2.4. Transmission Electron Microscopy

The isolated *E. coli*-OMVs obtained through ultracentrifugation were fixed with 2.5% glutaraldehyde. Afterwards, the vesicles were stained with 2% uranyl acetate for 30 s and examined with transmission electron microscopy (FEI TECNAI G2, Hillsborough, OR, USA).

### 2.5. Nanoparticle Tracking Analysis

The obtained *E. coli*-OMVs were quantitatively diluted to 1 mL with PBS, vortexed to ensure uniform distribution, and subjected to particle size analysis using NanoSight NS300 (Malvern Panalytical, Malvern, UK).

### 2.6. Labeling of E. coli-OMVs

To examine internalization of *E. coli*-OMVs by CT26 cells, *E. coli*-OMVs were first labeled by PKH67 (Umibio, Shanghai, China) (extracellular vesicle green fluorescent labeling dye). PKH67 working solution was prepared according to the manufacturer’s protocol and used to stain vesicles for 10 min in the dark. CT26 cells were treated with labeled vesicles for 0, 6, 12 and 24 h and Hoechst was added to stain the nuclei for 10 min. The cellular uptake of OMVs was visualized under the confocal microscopy. (Andor Dragonfly, Oxford, UK).

### 2.7. Cells Viability Assay

A total of 1 × 10^4^ CT26 cells per well were seeded in a 96-well plate. The cells were administrated with different *E. coli*-OMVs concentrations of 0, 5, 10, 20 and 40 μg/mL for 24 and 48 h at 37 °C. The Cell Counting Kit-8 (CCK8, Solarbio, China) (cell proliferation and cytotoxicity assay kit) was added to each well and incubated at 37 °C in the dark for 1 h. Absorbance at the wavelength of 450 nm was measured using a microplate reader (Bio-Rad 550, Hercules, CA, USA).

### 2.8. Cell Invasion and Migration Analysis

For the cell migration experiment, CT26 were seeded in a 6-well plate. Subsequently, serum-free medium containing *E. coli*-OMVs at protein concentrations of 0, 5, 10, and 20 μg/mL was added. The cell migration ability was detected by a scratch experiment (The cells at the edge of the scratch will gradually enter the blank area to heal the “scratch”). Cell invasion was detected by Transwell experiment. 150 μL of serum-free medium containing different concentrations of *E. coli*-OMVs was added to the upper chamber, and 700 μL of serum-containing medium was added to the lower chamber. After co-incubation at 37 °C for 24 h, the cells were fixed with 4% paraformaldehyde, and the bottom of the chambers was stained with 0.1% crystal violet. The stained cells were observed using an inverted microscope (Leica Microsystems, Wetzlar, Germany), and five random fields were selected for cell counting and statistical analysis.

### 2.9. Annexin V-FITC/PI Flow Cytometry Analysis

The cells were inoculated in a 6-well plate and treated with *E. coli*-OMVs at concentrations of 0, 5, 10 and 20 μg/mL for 48 h. Subsequently, both the supernatant and adherent cells were collected, centrifuged at 1, 000 g for 5 min, washed twice with PBS, stained with Annexin V and Propidium Iodide (Elabscience, E-CK-A211) according to the manufacturer’s protocol, and the apoptotic rate (the sum of early apoptosis rate and late apoptosis rate) for each group was determined using the flow cytometer (Beckman CytoFLEX, Brea, CA, USA).

### 2.10. Mitochondrial Membrane Potential Assay

The cells were inoculated in a 12-well plate and treated with *E. coli*-OMVs at concentrations of 0, 5, 10 and 20 μg/mL for 48 h. For each group, 500 μL of culture medium was added, followed by the addition of 500 μL of 1 × JC-1 working solution. Incubation was conducted at 37 °C in the dark. The color transition and change ratio of JC-1 fluorescence were detected by flow cytometry (Beckman CytoFLEX, Kraemer Blvd. Brea, CA, USA) immediately.

### 2.11. Western Blot

CT26 cells were treated with *E. coli*-OMVs at protein concentrations of 0, 5, 10, and 20 μg/mL for 48 h. CT26 cells were lysed in RIPA lysis buffer and total cellular protein was obtained. The protein concentrations of both cells and undissolved vesicles were determined using the BCA protein assay kit. Total proteins were separated by electrophoresis SDS-PAGE gel and transferred to PVDF membranes. The membrane was blocked with BSA (5% *w*/*v*; TBST) at room temperature for 1 h, followed by three washes with TBST, each for 10 min. Subsequently, the membrane was incubated overnight at 4 °C with primary antibodies, including OmpA (Cat #: 111120, 1:5000, Antibody Research Corporation, Saint Peters, NJ, USA), OmpC (BS-20213R, 1:5000, Thermo Fisher Scientific, Waltham, MA, USA), Bax (ab32503, 1:5000, Abcam), Bcl-2 (ab182858, 1:2000, Abcam) and β-actin (AF5003, 1:2000, Beyotime). After washing the PVDF membrane with TBST, it was further incubated with the secondary antibody (ab205718, 1:10,000, Abcam) at room temperature for 1 h. Protein bands were visualized using an ECL kit. Normalization of protein expression levels was performed based on β-actin expression, and Image J software (version 1.46, National Institutes of Health, Bethesda, MD, USA) was employed for analysis.

### 2.12. Xenograft Experiment In Vivo

Female BALB/c mice at 6 weeks of age were raised in a specified SPF-level environment and provided with sterile food and water. All mice were subcutaneously injected with CT26 cells (5 × 10^6^). When the tumor volume reached around 100 mm^3^, all mice were randomly divided into 4 groups, each consisting of 5 mice. Intratumoral injections of PBS (Control group) and *E. coli*-OMVs (low-dose OMVs group: 0.25 mg/kg, medium-dose OMVs group: 0.5 mg/kg, and high-dose OMVs group: 1 mg/kg) were administered every two days for a total of five times. Prior to injections, calipers were used to measure the long and short diameters of the tumor tissue. Tumor volume was calculated using the formula $v=\frac{\pi }{6}a\times b^{2}$ (*a* = long diameter, *b* = short diameter). Mouse weights were measured using an electronic scale. The mice were euthanized 48 h after the final injection, and after removing the tumor tissue, further processing and observation were conducted.

### 2.13. Immunohistochemistry

Tissue sections were deparaffinized in xylene and rehydrated in alcohol. The detailed procedures were described in a previous study [12]. Antigen retrieval was performed using a microwave repair method. After cooling the sodium citrate solution to room temperature, the sections were incubated overnight at 4 °C with primary antibodies (Bax: 1:500; Bcl-2: 1:250). Subsequently, they were incubated with a secondary antibody coupled with horseradish peroxidase at 37 °C for 30 min, followed by a 15 min incubation with a reaction amplifier. DAB staining was performed for 5 min, and counterstaining with hematoxylin was carried out. After dehydration, the sections were observed under a fluorescence microscope (Leica Microsystems, Germany).

### 2.14. Data Analysis

All data are presented as the mean ± standard deviation. GraphPad Prism 9.5.0 (San Diego, CA, USA) was used for data analysis. Statistical significance between the control group and experimental groups was assessed using one-way or two-way ANOVA. Differences between control group and experimental groups were considered statistically significant when *p* < 0.05 (* *p* < 0.05, ** *p* < 0.01, *** *p* < 0.001). The results were not statistically significant when *p* > 0.05 (ns).

## 3. Results

### 3.1. Characterization of E. coli-OMVs

**Figure 1:**
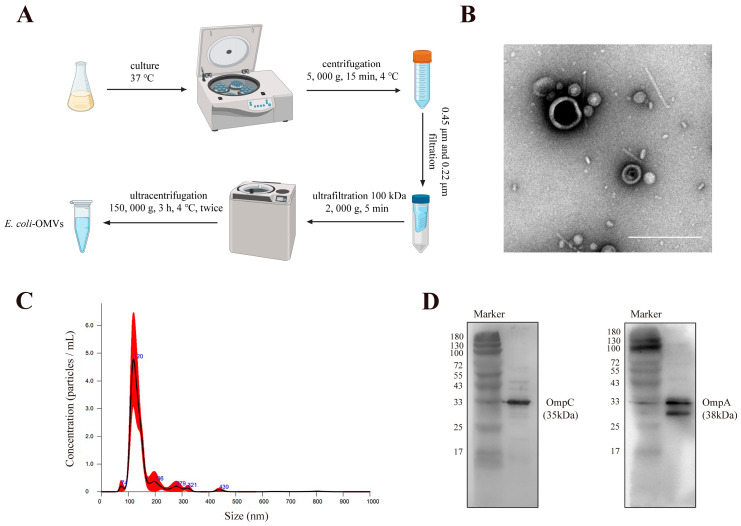
Characterization of *E. coli*-OMVs. (**A**) Extraction of *E. coli*-OMVs. The image was created using BioRender (www.biorender.com) (**B**) Transmission electron microscope images of purified *E. coli*-OMVs. Scale bar: 200 nm. (**C**) Nanoparticle tracing analysis of *E. coli*-OMVs. (**D**) Western blot analysis was obtained for the presence of two characteristic proteins of *E. coli*-OMVs. All experiments were repeated three times at least.

OMVs were isolated from the culture supernatant of *E. coli* using ultracentrifugation (Figure 1A). Transmission electron microscopy was employed to observe the morphological characteristics of the *E. coli*-OMVs, revealing a bilayer lipid membrane structure. (Figure 1B). Nanoparticle tracking analysis indicated that the average diameter of the OMVs was (149.6 ± 10.0) nm (Figure 1C). Western blot results demonstrated the presence of the *E. coli* outer membrane characteristic proteins OmpA (38 kDa) and OmpC (35 kDa) in *E. coli*-OMVs (Figure 1D). These findings collectively confirm the isolated vesicles were *E. coli*-OMVs.

### 3.2. E. coli-OMVs Inhibit CT26 Colon Cancer Growth In Vivo

**Figure 2:**
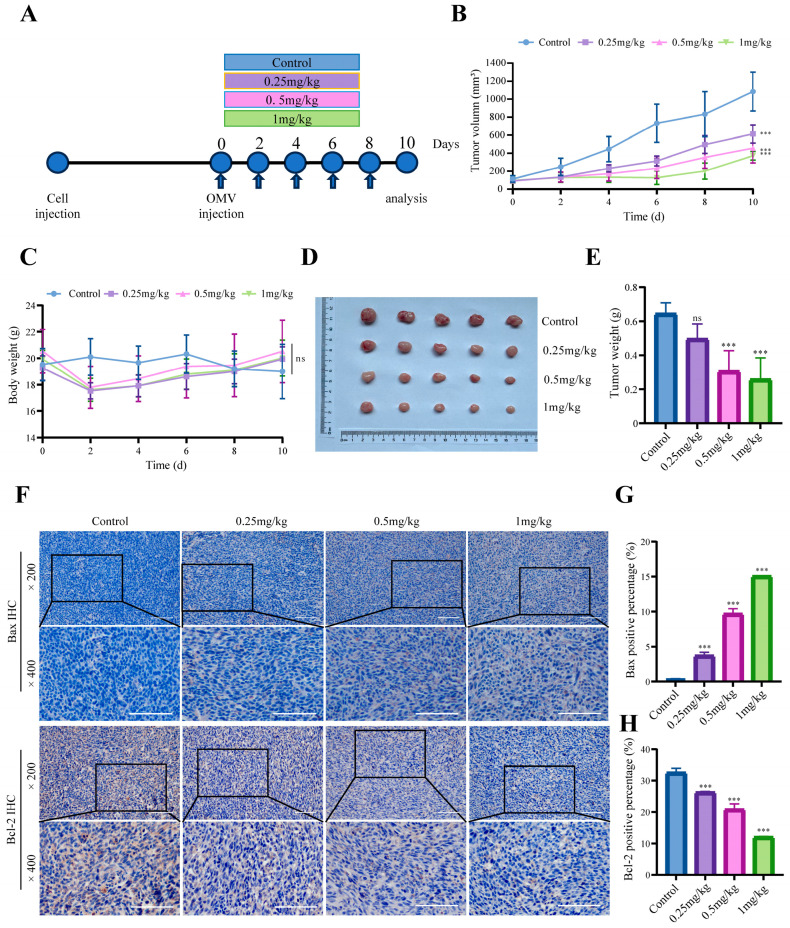
The effect of *E. coli*-OMVs on the growth of CT26 tumor-bearing mice. (**A**) The schematic diagram of injection. (**B**) The growth curve of the transplanted tumor volume in each group of mice. (**C**) Body weight change curve of mice during overall period. (**D**) Comparison of the morphological characteristics. (**E**) and weight of the tumor xenograft in the four groups of mice. (**F**) Immunohistochemical staining of tumor tissues and. Scale bar: 100 μm. (**G**,**H**) analysis of the expression levels of Bax and Bcl-2. *p* > 0.05 (ns), *** *p* < 0.001. All experiments were repeated three times at least.

To explore the impact of vesicles on colorectal cancer, we conducted in vivo experiments of heterotopic tumor transplantation in BALB/c mice. Tumor-bearing mice were randomly divided into four groups, each receiving injections of different doses of OMVs every two days. Tumor size and body weight of tumor-bearing mice were monitored before injections (Figure 2A). The results showed an increasing trend in tumor size in different groups, and compared to the control group, the tumor growth in the OMVs treatment groups was slower (Figure 2B). There was no statistical difference in the average weight of mice among the four groups before the injections (*p* > 0.05). After the first injection for two days, the average weight of mice in the OMVs groups decreased, gradually increasing after the third injection (Figure 2C). After 10 days, the mice were euthanized, and heterotopic transplants were removed for evaluation of tumor tissue weight. The OMVs groups exhibited a significant reduction in tumor tissue weight compared to the control group (Figure 2D,E).

Subsequently, we then examined whether *E. coli*-OMVs could inhibit the growth of CT26 colon cancer in vivo by promoting apoptosis. We used immunohistochemistry to study the expression of the pro-apoptotic protein Bax and the anti-apoptotic protein Bcl-2 in tumor tissues. Immunohistochemical results showed an increase in Bax expression and a decrease in the anti-apoptotic protein Bcl-2 expression in the OMVs groups (Figure 2F–H). The above results indicate that *E. coli*-OMVs can inhibit the growth of xenograft CT26 colon cancer in mice and promote their apoptosis.

### 3.3. E. coli-OMVs Enter CT26 Cells and Inhibit Cell Viability

**Figure 3:**
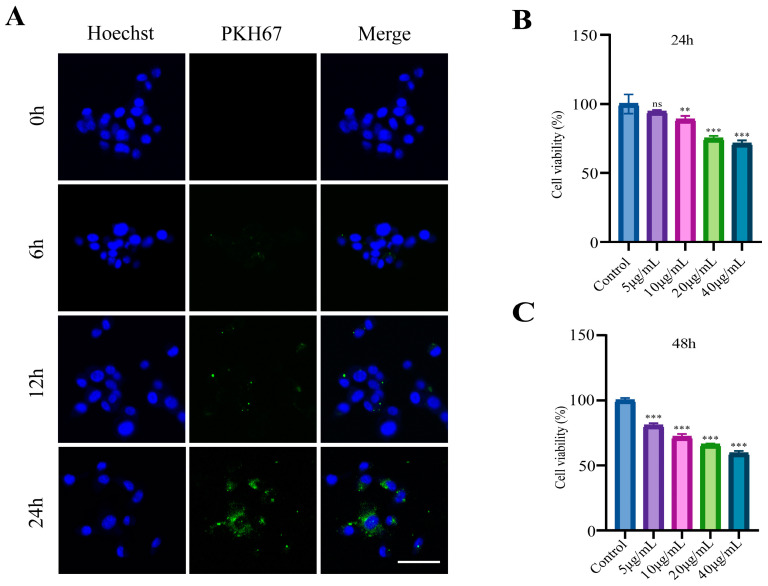
*E. coli*-OMVs entered CT26 and reduced the cell viability. (**A**) Fluorescence images of *E. coli*-OMVs taken up by CT26. Scale bar: 100 μm. (**B**,**C**) After being treated with *E. coli*-OMVs at 24 and 48 h, compared with the control group, the cell viability of CT26 significantly decreased. *p* > 0.05 (ns), ** *p* < 0.01, *** *p* < 0.001. All experiments were repeated three times at least.

Fluorescently labeled *E. coli*-OMVs were co-incubated with CT26 colorectal cancer cells for 0, 6, 12 and 24 h. Fluorescence images revealed green fluorescent labeled OMVs in the cytoplasm and around the cell nucleus of CT26 cells (Figure 3A), indicating the uptake of *E. coli*-OMVs by CT26 cells. Subsequently, to verify the impact of *E. coli*-OMVs on colorectal cancer cells, the CCK8 method was employed to measure the OD values of CT26 cells treated with different concentration of *E. coli*-OMVs (0, 5, 10 and 20 μg/mL) at 24 and 48 h, calculating cell viability. Compared to the control group, all groups showed a decreasing trend (Figure 3B,C).

### 3.4. E. coli-OMVs Inhibited the Migration and Invasion of CT26 Cells

**Figure 4:**
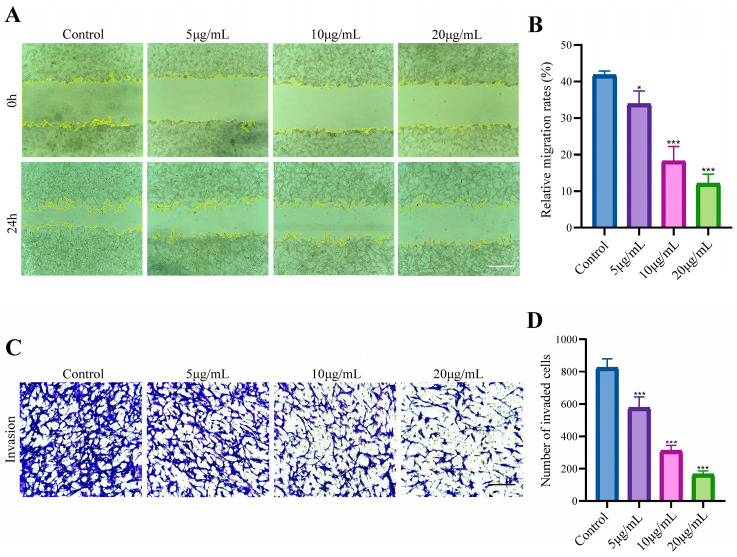
*E. coli*-OMVs inhibited the migration and invasion of CT26 cells. (**A**) *E. coli*-OMVs inhibited the migration of CT26 cells. Scale bar: 520 μm. (**B**) The relative migration rates of CT26 cells. (**C**) *E. coli*-OMVs inhibited the invasion of CT26 cells. Scale bar: 210 μm. (**D**) The number of invaded CT26 cells in each group. * *p* < 0.05, *** *p* < 0.001. All experiments were repeated three times at least.

Scratch experiments was used to verify the effect of vesicles on the migration of CT26 cells. *E. coli*-OMVs slowed down the cell movement to the middle scratch site (Figure 4A,B). The invasion effect of *E. coli*-OMVs treated cells after 24 h was detected by Transwell assays. *E. coli*-OMVs reduced the invasion number of lower compartment cells (Figure 4C,D).

### 3.5. E. coli-OMVs Promoted Apoptosis of CT26 Cells

**Figure 5:**
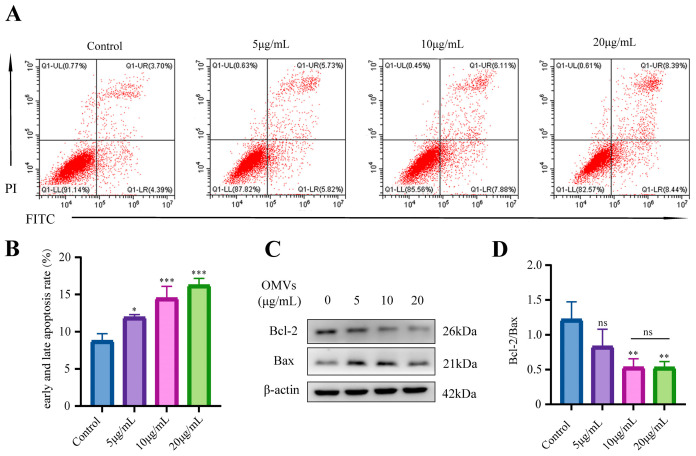
*E. coli*-OMVs promoted apoptosis of CT26 cells. (**A**) CT26 cells were treated with *E. coli*-OMVs for 48 h. The cells were stained with Annexin V and PI, and the number of apoptotic cells was determined by flow cytometry. (**B**) The rate of early and late apoptosis was calculated (early apoptosis: Q1-LR, late apoptosis: Q1-UR). (**C**) Changes in Western Blotting of Bax, Bcl-2 after *E. coli*-OMVs act on CT26 cells. β-actin was used as a control. (**D**) The ratio of Bcl-2 to Bax was presented. *p* > 0.05 (ns), * *p* < 0.05, ** *p* < 0.01, *** *p* < 0.001. All experiments were repeated three times at least.

We used Annexin V/PI double staining to detect the effect of *E. coli*-OMVs on the apoptosis of CT26 cells. When cells were treated with different concentrations of *E. coli*-OMVs for 48 h, the early and late apoptosis rates in the OMVs groups increased with the concentration compared to the control group, indicating that *E. coli*-OMVs promote apoptosis of CT26 cells (Figure 5A,B). The Bcl-2 family mainly includes the anti-apoptotic protein Bcl-2 and the pro-apoptotic protein Bax, and the ratio between them has been confirmed to reflect the tendency of cell apoptosis. We measured changes in the protein levels of Bcl-2/Bax. The Western blot results reveal a progressive reduction in the Bcl-2/Bax ratio with increasing concentration of *E. coli*-OMVs, with no significant difference between 10 μg/mL *E. coli*-OMVs group and 20 μg/mL *E. coli*-OMVs group (Figure 5C,D). These results suggest that *E. coli*-OMVs can induce apoptosis in CT26 cells.

## 4. Discussion

The role of bacteria in the treatment of CRC has been confirmed and has attracted widespread attention. However, effective and safe therapeutic approaches still require further investigation [16]. OMVs are products derived from bacteria, and since most vesicles are produced through budding, they contain outer membrane proteins and periplasmic contents from the parental bacteria [17,18]. Previous studies have shown that OMVs are engineered and used as drug carriers to exhibit similar characteristics to parental bacteria in tumor targeting and treatment as well as immune activation [18,19,20,21,22,23,24]. However, few studies have explored the direct impact of OMVs themselves on tumors.

We extracted *E. coli*-OMVs by differential centrifugation, but it may still be impossible to obtain pure OMVs because in the existing extraction technology, whether it is differential centrifugation or density gradient centrifugation, non-EV nanoparticles may still exist in the extract. This will be the direction we need to further improve in the future. We characterized OMVs by NTA, WB, and TEM to explore the basic characteristics of *E. coli*-OMVs. TEM results show that the average diameter of vesicles may be approximately 50 nm, which is inconsistent with NTA results. NTA detection missed the very small vesicles (<50 nm) visible on TEM images and vesicle dehydration in TEM experiments. Subsequently, OMVs were found to inhibit CT26 colorectal cancer growth in vivo by measuring tumor volume and weight of mice. In addition, we found that *E. coli*-OMVs can penetrate into the intracellular space of CT26 cells and inhibit their proliferation, invasion, and migration. In an experiment assessing the impact of *E. coli*-OMVs on mouse body weight, we observed a non-significant decrease in body weight after *E. coli*-OMVs injection compared to the control group. We believe that the safe dosage of *E. coli*-OMVs and their effects on the organism require further experimental exploration. Although our study demonstrates the inhibitory effect of *E. coli*-OMVs on the growth of CT26 colorectal cancer both in vitro and in vivo. However, the anti-tumor function and molecular mechanisms of *E. coli*-OMVs still require further extensive research.

Tumor growth is characterized by an imbalance between cell proliferation and apoptosis, which in turn leads to abnormal cell proliferation [25]. A previous study about the treatment of colorectal tumors with *E. coli*-OMVs has shown that transcriptomic analysis reveals an increase in apoptosis-related genes, providing crucial clues to explore the anti-tumor mechanisms of *E. coli*-OMVs [20]. Therefore, in cancer treatment, effectively inhibition of tumor growth can be achieved by directing cancer cells towards the apoptotic pathway [26]. In our study, flow cytometry demonstrated that *E. coli*-OMVs induces an increase in the rate of early and late apoptosis and a decrease in mitochondrial membrane potential in CT26 cells (Figure S1). The decrease in mitochondrial membrane potential suggests that *E. coli*-OMVs may induce apoptosis through mitochondria-dependent pathways.

The regulation of cell apoptosis mainly involves death receptor-mediated pathways and mitochondria-mediated pathways [27]. The Bcl-2 family mainly includes the anti-apoptotic protein Bcl-2 and the pro-apoptotic protein Bax, and the ratio between them has been confirmed to reflect the tendency of cell apoptosis [28]. Our research results show that *E. coli*-OMVs reduce the ratio of Bcl-2/Bax. However, inducing cell mitochondrial apoptosis involves multiple signaling pathways and molecular mechanisms [29,30]. In addition to the regulation by the Bcl-2 family mentioned in this study, severe DNA damage and the accumulation of reactive oxygen species (ROS) are also common triggers for mitochondrial apoptosis [31,32,33,34]. These aspects need to be further explored to fully understand the mechanism of action of *E. coli*-OMVs in anti-tumor therapy.

Bacterial extracellular vesicles could play an important role in the interaction between the microorganism and the host [35]. EHEC O157 OMVs carry key virulence factors including Shiga toxin 2a (Stx2a), cytolethal distending toxin V (CdtV), EHEC hemolysin, and flagellin, thereby causing cell death [36]. OMV-associated EHEC-Hly triggers the mitochondrial apoptotic pathway in human microvascular endothelial and intestinal epithelial cells [37]. However, which contents from the outer membrane vesicles exert anti-tumor effects has not been reported. Previous studies have indicated that *E. coli* l-asparaginase possesses anti-tumor activity [38]. Additionally, soluble recombinant endostatin purified from *E. coli* demonstrates both anti-angiogenic and anti-tumor properties [39]. Since OMVs contain parental substances, we speculate that the antineoplastic substances present in *E. coli* are also present in OMVs and play a similar role. This opens up new avenues for future in-depth studies, allowing researchers to have a more comprehensive understanding of the mechanisms by which *E. coli*-OMVs function in anti-tumor therapy.

## Data Availability

The original contributions presented in the study are included in the article/Supplementary Materials, further inquiries can be directed to the corresponding author.

## Supplementary Materials

The following supporting information can be downloaded at: https://www.mdpi.com/article/10.3390/microorganisms12071446/s1, Figure S1: The effect of *E. coli*-OMVs on mitochondrial membrane potential of CT26.
