# Supplementary material for: Extracellular Membrane Vesicles of Escherichia coli Induce Apoptosis of CT26 Colon Carcinoma Cells
Source: Microorganisms. 2024 Jul 17;12(7):1446. doi: 10.3390/microorganisms12071446 (PMC11279139; doi:10.3390/microorganisms12071446)
Supplement: Supplementary file 1 [file microorganisms-12-01446-s001.zip › microorganisms-2806986-supplementary.pdf]

Supplementary figure S1

We studied the effects of *E. coli*-OMVs on the mitochondrial membrane potential of CT26 cells. We measured the fluorescence shift (from red to green) of JC-1 dye sensitive to △ψm in CT26 cells. Compared to the control group, the cells treated with 10 and 20μg/mL *E. coli*-OMVs showed a significant decrease in the ratio of aggregates to monomers, indicating a reduction in mitochondrial membrane potential (Figure S1).

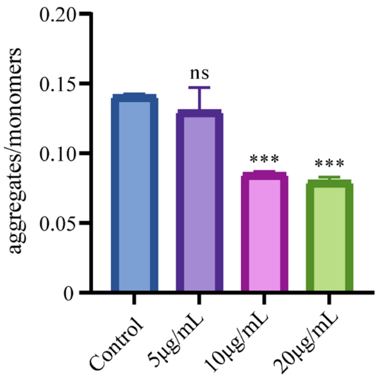

Figure S1. CT26 cells were treated with various concentrations of *E. coli*-OMVs for 48 h and then stained with JC-1. Calculate the ratio of aggregates to monomers according to the flow results. All experiments were repeated three times at least. (***$p < 0.001$, ns = non-significant)
